# Concealed Wolff–Parkinson–White Syndrome revealed by acute coronary syndrome

**DOI:** 10.1111/anec.12735

**Published:** 2019-12-19

**Authors:** Ricardo Lessa de Castro, Neiberg de Alcantara Lima, Danielli Oliveira da Costa Lino, Susan Faragher Bannon

**Affiliations:** ^1^ Department of Internal Medicine Homer Stryker M.D. School of Medicine Western Michigan University Kalamazoo MI USA; ^2^ Department of Cardiology Universidade Estadual do Ceara Fortaleza Brazil

**Keywords:** acute myocardial infarct, coronary artery disease, Parkinson, White, Wolff

## Abstract

Wolff–Parkinson–White (WPW) syndrome is a conduction disturbance in which atrial impulses are transmitted to the ventricles by an accessory pathway instead of the normal atrioventricular conduction. The WPW syndrome may either simulate myocardial infarction or mask the electrocardiographic abnormalities of an acute MI. However, concealed WPW revealed after an acute coronary syndrome is rare with few cases reported in the literature. This article reports a case of coronary artery disease with ST‐segment elevation in a 57‐year‐old man, previously asymptomatic, with an initial electrocardiogram showing no conduction abnormalities that subsequently presented with an ECG compatible with WPW.

## 1 INTRODUCTION

Wolff–Parkinson–White (WPW) syndrome is a conduction disturbance in which atrial impulses are transmitted to the ventricles by an accessory pathway instead of the normal atrioventricular conduction. The result of these multiple fronts of depolarization is a short PR interval, the delta wave, and a widened QRS complex. Patients with WPW typically present with palpitations, syncope, and sudden death (Wolff, Parkinson, & White, 1930). Increased sympathetic tone because of physical exercise or chest pain can enhance conduction over the normal atrioventricular conduction system (Astorri & Pattoneri, 2006). The WPW syndrome may either simulate myocardial infarction or mask the electrocardiographic abnormalities of an acute MI (Renguang & Qinghua, 2013). However, concealed WPW revealed after an acute coronary syndrome is rare with few cases reported in the literature. This article presents a case of WPW revealed after an ST‐elevation myocardial infarct with no previous abnormalities in his electrocardiogram (EKG).

## 2 CASE PRESENTATION

A 57‐year‐old man with a past medical history significant for diabetes and hypertension presented to ED with nonradiating anterior chest pain that started at rest, with onset 4 hr before his presentation. The chest pain was associated with nausea and vomiting. He endorsed prior episodes of chest pain, but it was self‐limited and lower in intensity.

Social history was negative for drug and alcohol use. He denied palpitations, dizziness, and lightheadedness. His home medications included losartan 50 mg daily with carvedilol 25 mg two times a day and metformin 500 mg three times a day.

The physical examination was remarkable only for mild to moderate distress due to chest pain. The vital signs were normal with blood pressure at presentation of 135 over 90 mmHg, oxygen saturation 98% on ambient air, and heart rate 85 beats per minute. The heart and lung sounds were normal with pulses palpable and symmetrical.

**Figure 1:**
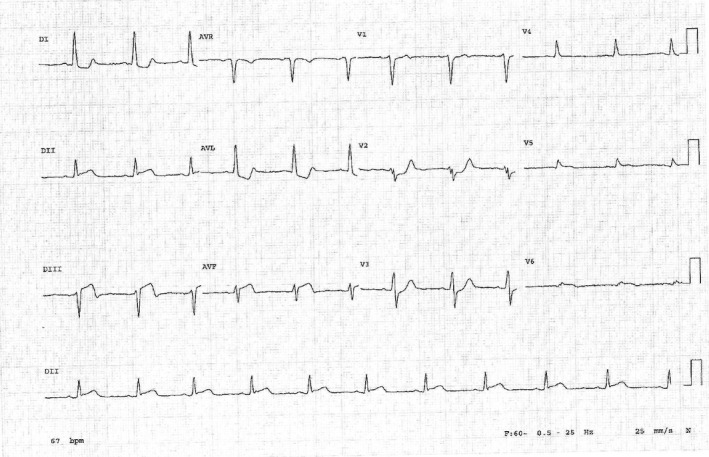
Inferior heart infarct with no PR abnormalities

At the emergency department, he had an EKG done which showed a left axis with sinus rhythm, normal PR interval, and narrow QRS complexes. There was ST elevation in leads II, III, aVF, V5, and V6 (Figure 1) with depression in leads I, aVL, and V2. His initial troponin was elevated at 0.071 ng/ml (normal range 0.0 to 0.025 ng/ml), hemoglobin normal at 13.4 g/dl, and creatinine mildly elevated at 1,26 mg/dl.

A diagnosis of ST‐elevation myocardial infarct (STEMI) was made, and the patient was started on morphine, aspirin, and clopidogrel and sent immediately to heart catheterization where he had a drug‐eluting stent placed in his occluded right coronary artery.

**Figure 2:**
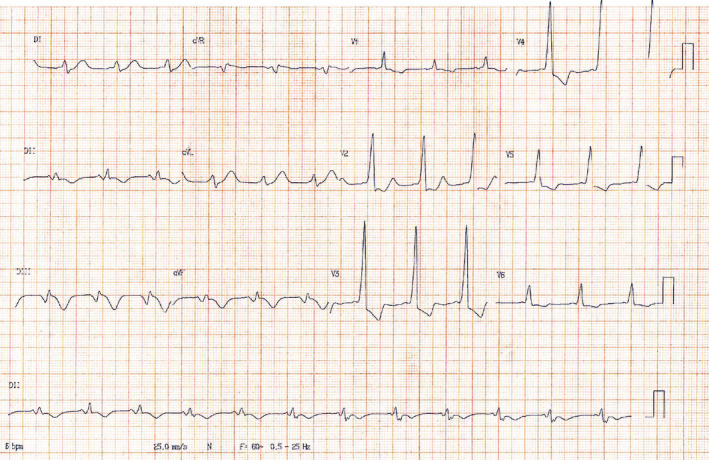
WPW pattern after stent angioplasty

A postprocedure EKG showed negative T waves in the inferior leads and a surprising short PR interval with a wide QRS complex with a slurred onset of the QRS waveform in the early part of QRS compatible with delta wave of Wolff–Parkinson–White (Figure 2).

The remainder of the hospitalization was uneventful, and he was discharged home asymptomatic for further outpatient treatment on aspirin, clopidogrel, lisinopril, atorvastatin, and carvedilol.

In his third‐, sixth‐, and twelfth‐month follow‐ups, he had no complaints. Patient deferred further studies or procedure for his WPW.

## 3 DISCUSSION

In 1930, Dr. Louis Wolff, Sir John Parkinson, and Paul Dudley White described a case series of 11 patients with a syndrome that now bears their names (Scheinman, 2005).

Usually, WPW is asymptomatic. In adults, after the fourth decade of life, symptoms can occur (Rosenfeld, Zetta, & Batsford, 1991) but they do so much less frequently compared with earlier decades. However, WPW syndrome is associated with a small but lifetime risk of catastrophic events and sudden cardiac death (Pappone et al., 2012).

Wolff–Parkinson–White (WPW) syndrome may either simulate myocardial infarction (MI) or mask the electrocardiographic abnormalities of acute MI (Renguang & Qinghua, 2013). Reports on the appearance of the WPW syndrome after acute myocardial infarction are notably sparse (Goel & Han, 1974). As to latent WPW syndrome, bypass tract's refractory period is longer than sinus PP interval or the normal A–V conduction system which overtakes the conduction from the accessory bundle, leading to no pre‐excitation. There can be several possible mechanisms for the appearance of the WPW syndrome following myocardial infarction. (Goel & Han, 1974). The ischemia or infarction either shortening the bypass tract's refractory period, prolonging sinus PP interval or prolonging AV nodal conduction time, can induce typical EKG manifestations of WPW.

Similar to our case, M.G. Kaya et al. described a 60‐year‐old woman with signs of anterior ischemia and a heart catheterization showing a lesion in the proximal left anterior descending artery (LAD) which was later treated with stent placement (Kaya et al., 2008).

Our report shows the rare finding of a new WPW uncovered by an acute coronary syndrome in an asymptomatic patient with a previously normal EKG.

## 4 CONCLUSION

WPW is a conduction system abnormality that can be asymptomatic or cause symptoms that can be as mild as palpitations or fatal as sudden death. Some patients have concealed WPW which can be revealed in specific circumstances such as myocardial ischemia.

In the setting of ST elevation infarction, WPW should be part of the differential diagnosis as in WPW, the EKG can mimic cardiac ischemia.

It is rare and uncommon for myocardial infarction or ischemia to uncover a concealed WPW. This diagnosis can impact the overall treatment of acute coronary ischemia because beta‐blockers may be contraindicated.

## CONFLICT OF INTEREST

The authors declare that they have no conflicts of interest with the content of this case report.

## AUTHORS’ CONTRIBUTION

All authors reviewed and approved the manuscript. Collected patient data: Ricardo de Castro, Danielli Lino. Wrote the main manuscript: Ricardo de Castro, Susan Bannon. Gave suggestions on this manuscript: Neiberg Lima, Danielli Lino.

## ETHICS

This case report was writing respecting patient confidentiality and privacy. Patient had the opportunity to read the present case report and had no objections to the final abstract.
